# Development and external validation of nomograms in oropharyngeal cancer patients with known HPV-DNA status: a European Multicentre Study (OroGrams)

**DOI:** 10.1038/s41416-018-0107-9

**Published:** 2018-05-24

**Authors:** Christian Grønhøj, David H. Jensen, Christian Dehlendorff, Linda Marklund, Steffen Wagner, Hisham Mehanna, Eva Munck-Wikland, Torbjörn Ramqvist, Anders Näsman, Claus Wittekindt, Nora Würdemann, Shachi Jenny Sharma, Stefan Gattenlöhner, Katalin Kiss, Elo Andersen, Rachel Spruce, Nikos Batis, Max Robinson, Kevin Harrington, Stuart Winter, Terence M. Jones, Jens Peter Klussmann, Tina Dalianis, Jeppe Friborg, Christian von Buchwald

**Affiliations:** 1 Department of Otorhinolaryngology, Head and Neck Surgery and Audiology, Rigshospitalet, University of Copenhagen, Copenhagen, Denmark; 2 Statistics and Pharmacoepidemiology, Danish Cancer Society Research Center, Copenhagen, Denmark; 3 Department of Clinical Science and Technology (CLINTEC), Karolinska Institutet, Stockholm, Sweden; 4 Department of Otorhinolaryngology, Head and Neck Surgery, University of Giessen, Giessen, Germany; 5 Head and Neck Studies and Education (InHANSE), University of Birmingham, Birmingham, UK; 6 Department of Oncology-Pathology, Karolinska Institutet, Stockholm, Sweden; 7 Department of Pathology, University of Giessen, Giessen, Germany; 8 Department of Pathology, Rigshospitalet, University of Copenhagen, Copenhagen, Denmark; 9 Department of Oncology, Herlev Hospital, University of Copenhagen, Copenhagen, Denmark; 10 Centre for Oral Health Research, Newcastle University, Newcastle, UK; 11 The Institute of Cancer Research/The Royal Marsden NIHR Biomedical Research Centre, London, UK; 12 Department of Otorhinolaryngology, Head and Neck Surgery, Nuffield Department of Medicine, Oxford, UK; 13 Institute of Translational Medicine, University of Liverpool, Liverpool, UK; 14 Department of Oncology, Rigshospitalet, University of Copenhagen, Copenhagen, Denmark

**Keywords:** Head and neck cancer, Epidemiology

## Abstract

**Background:**

The proxy marker for human papillomavirus (HPV), p16, is included in the new AJCC 8th/UICC 8th staging system, but due to incongruence between p16 status and HPV infection, single biomarker evaluation could lead to misallocation of patients. We established nomograms for overall survival (OS) and progression-free survival (PFS) in patients with oropharyngeal squamous cell carcinoma (OPSCC) and known HPV-DNA and p16 status, and validated the models in cohorts from high- and low-prevalent HPV countries.

**Methods:**

Consecutive OPSCC patients treated in Denmark, 2000–2014 formed the development cohort. The validation cohorts were from Sweden, Germany, and the United Kingdom. We developed nomograms by applying a backward-selection procedure for selection of variables, and assessed model performance.

**Results:**

In the development cohort, 1313 patients, and in the validation cohorts, 344 German, 503 Swedish and 463 British patients were included. For the OS nomogram, age, gender, combined HPV-DNA and p16 status, smoking, T-, N-, and M-status and UICC-8 staging were selected, and for the PFS nomogram the same variables except UICC-8 staging. The nomograms performed well in discrimination and calibration.

**Conclusions:**

Our nomograms are reliable prognostic methods in patients with OPSCC. Combining HPV DNA and p16 is essential for correct prognostication. The nomograms are available at www.orograms.org.

## Introduction

In most parts of the Western world, the main risk factor for oropharyngeal squamous cell carcinoma (OPSCC) is now infection with high-risk human papillomavirus (HPV); while, a smaller proportion is related to a high consumption of alcohol and smoking tobacco.^1–4^ Patients with HPV-associated OPSCCs have improved survival probably related to a different mutational profile,^5,6^ immune response^7–9^ and clinical features.^10^

p16 overexpression is a proxy marker for HPV-driven carcinogenesis which is the main prognostic factor in patients with OPSCC. Consequently, p16 was included in the newly proposed American Joint Committee on Cancer/Union for International Cancer Control (AJCC-8/UICC-8) staging system. However, an estimated 10–20% of all OPSCCs are p16-positive, but HPV-negative, due to alternative cellular events leading to p16 overexpression^11,12^ being most apparent in oropharyngeal non-tonsillar, non-base of tongue cancer.^13^ Hence, it may be suboptimal to stratify patients based on evaluation of a single biomarker (i.e. p16 alone) due to the risk of misclassification of tumours and thereby misallocation of patients with an undesired prognosis.^14,15^ The combination of HPV-DNA and p16 status has shown better prognostication.^16^ Available nomograms so far for patients with OPSCC do not include combined HPV-DNA and p16 status, and models have not been externally validated across areas with high and low HPV prevalence.^17,18^

A nomogram is a graphical illustration of a statistical model for calculating the cumulative effect of several variables on a particular outcome, and nomograms have been developed to predict clinical endpoints for patients with several types of malignancies. In this study, we aimed to identify OPSCC- and patient-related factors associated with OS and PFS, and to construct and externally validate predictive nomograms. Moreover, this is the first study addressing patients treated for an OPSCC encompassing high and low HPV-prevalent countries in validation cohorts, and incorporating the newly published AJCC-8/UICC-8 staging system refining prognostication by employing both HPV-DNA and p16 status.

## Materials and methods

### Patient cohorts and determination of p16 overexpression and presence of HPV DNA

#### The development cohort

Consecutive patients diagnosed with OPSCC and treated with curative intent in eastern Denmark between 2000 and 2014 were included in the development cohort.^1,19,20^ Using the unique resident code from the Danish Civil Registration System, we linked the Danish Head and Neck Cancer Group (DAHANCA)^21^ database and the Danish Pathology Data Registry (DPDR),^22^ to identify patients. Patient characteristics were retrieved from these databases as well as from medical records. Curative radiotherapy regimens consisted of 66–68 Gy, divided into 33–34 fractions given 6 days a week. From 2007, stage III–IV (UICC 7th) patients were offered concurrent chemotherapy (primarily weekly cisplatin 40 mg/sqm), if tolerated, whilst a minority were treated with cetuximab.

An expert head-and-neck pathologist re-validated a haematoxylin–eosin (H&E)-stained section of each tumour. p16 staining was considered positive if there was a strong and diffuse nuclear and cytoplasmic reaction in more than 70% of the tumour cells.^23^ Immunohistochemistry for p16 was performed using the Ventana Benchmark Ultra autostainer with the UltraView detection kit and the p16 monoclonal antibody E6H4 ready-to-use with CC1 as a pretreatment (Roche, Tuscon, USA).

DNA was isolated from two to four 10-μm sections using the DSP DNA Mini Kit and the QIAsymphony SP kit (Qiagen, Hilden, Germany), according to the manufacturers’ instructions. HPV DNA PCR was performed using the general primers GP5+/6+ and Platinum Taq DNA polymerase (Invitrogen, Naerum, Denmark). All GP5+/6+ PCR negative samples were subject to a GAPDH (housekeeping gene) PCR to confirm DNA quality. HPV DNA amplicons were run on the QIAxcel Advanced System using the QX DNA Screening Gel (Qiagen, Hilden, Germany) according to the manufacturer’s instructions. The expected amplicon sizes were ~150 base pairs (bp) for GP5+/6+ and 200 bp for GAPDH. Negative samples were resolved on a 2.5% agarose gel stained with ethidium bromide to compare the sensitivity of the current assay with the standard. Approximately 50% of all samples were analysed by HPV PCR and the HPV+ cases were subsequently sequenced for HPV typing.^1^ The remaining samples were analysed by HPV PCR.^19,20^ The GP5+/GP6+ primers used^24^ are known to amplify at least 37 mucosal HPV types,^25^ namely 14 high-risk HPV types 16, 18, 31, 33, 35, 39, 45, 51, 52, 56, 58, 59, 66 and 68, and 23 low-risk HPV types 6, 11, 26, 34, 40, 42, 43, 44, 53, 54, 55, 57, 61, 70, 71, 72, 73, 81, 82/MM4, 82/IS39, 83, 84 and 89. The specific HPV types can then be identified by, e.g. sequencing of the resulting amplicons.^1^

#### The validation cohorts

Three independent cohorts formed the external validation cohorts. The populations consisted of patients with OPSCC treated with curative intent at Karolinska University Hospital (Stockholm, Sweden; 2005–2012), Giessen University Hospital (Giessen, Germany; 2000–2009) and The Predictr Consortium, United Kingdom (UK) (2001–2012).

The Swedish cohort was classified using p16 immunohistochemistry (clone JC8, dilution 1:100, Santa Cruz Biotechnology, CA, USA or clone E6H4, DakoCytomation A/S, Carpinteria, CA, USA) and high-risk HPV-DNA detection by a bead-based multiplex assay on a Magpix instrument (LUMINEX Inc., Austin, TX, USA).^9^ The Magpix instrument is known to amplify the 27 HPV types: HPV 6, 11, 16, 18, 26, 30, 31, 33, 35, 39, 42, 43, 44, 45, 51, 52, 53, 56, 58, 59, 66, 67, 68, 69, 70, 73 and 82.

The German cohort was classified using p16 immunohistochemistry (CINtec histology, Roche mtm laboratories) and high-risk HPV-DNA detection by PCR followed by bead-based hybridisation (Luminex Technology, Multimetrix, Progen, Heidelberg, Germany).^26^ This assay detects the following 24 HPV types: 16, 18, 31, 33, 35, 39, 45, 51, 52, 56, 58, 59, 68, 73 and 82, three putative high-risk types (26, 53 and 66) and six low-risk types (6, 11, 42, 43, 44 and 70).

The UK cohort was classified using p16 immunohistochemistry (CINtec histology, Roche mtm laboratories) and high-risk HPV DNA in situ hybridisation (Inform HPV III, Ventana Medical Systems Inc.).^3^ INFORM HPV III Family 16 probe B detects HPV-16, -18, -31, -33, -35, -39, -45, -51, -52, -56, -58 and 66.

Overexpression of p16 (>70% positive staining) was classified similar to the Danish cohort for all three validation cohorts. Treatment modalities for the validation cohorts are presented in more detail in Table 1.

**Table 1:** Patient characteristics in the four cohorts

Variable	Eastern Denmark	Giessen, Germany	Karolinska, Sweden	The Predictr Consortium, UK
Number of patients	1313	344	503	463
Overall survival (median [IQR])	3.62 [1.85, 5.00]	3.97 [1.18, 5.00]	5.00 [3.33, 5.00]	3.77 [1.73, 5.00]
Overall survival (%)				
Censored	888 (63.8)	164 (45.7)	389 (71.9)	420 (62.6)
Events	503	195 (54.3)	151 (27.9)	177 (26.4)
NA	0 (0.0)	0 (0.0)	1 (0.2)	74 (11.0)
Progression-free survial (median [IQR])	2.74 [1.12, 5.00]	2.48 [0.77, 5.00]	5.00 [2.46, 5.00]	3.59 [1.43, 5.00]
Progression-free survival (%)				
Censored	794 (57.1)	139 (38.7)	364 (67.3)	397 (59.2)
Events	545 (39.2)	220 (61.3)	174 (32.2)	203 (30.3)
NA	52 (3.7)	0 (0.0)	3 (0.6)	71 (10.6)
Smoking (%)				
Current	509 (38.8)	265 (77.0)	178 (35.4)	183 (39.5)
Former	532 (40.5)	40 (11.6)	164 (32.6)	156 (33.7)
Never	272 (20.7)	39 (11.3)	161 (32.0)	124 (26.8)
Male (%)	947 (72.1)	265 (77.0)	373 (74.2)	340 (73.4)
Age at diagnosis (median [IQR])	59.81 [53.93, 66.38]	58.89 [52.69, 64.97]	60.00 [53.00, 67.00]	56.00 [50.00, 63.00]
HPV-DNA and p16 status (%)				
HPV–/p16–	411 (31.3)	233 (67.7)	85 (16.9)	141 (30.5)
HPV–/p16+	84 (6.4)	23 (6.7)	27 (5.4)	31 (6.7)
HPV+/p16–	59 (4.5)	21 (6.1)	36 (7.2)	18 (3.9)
HPV+/p16+	759 (57.8)	67 (19.5)	355 (70.6)	273 (59.0)
*T* (%)				
T1	278 (21.2)	75 (21.8)	124 (24.7)	85 (18.4)
T2	614 (46.8)	98 (28.5)	178 (35.4)	184 (39.7)
T3	296 (22.5)	84 (24.4)	101 (20.1)	100 (21.6)
T4	125 (9.5)	87 (25.3)	100 (19.9)	94 (20.3)
*N* (%)				
N0	282 (21.5)	95 (27.6)	101 (20.1)	116 (25.1)
N1	686 (52.2)	47 (13.7)	94 (18.7)	77 (16.6)
N2	206 (15.7)	187 (54.4)	287 (57.1)	252 (54.4)
N3	139 (10.6)	15 (4.4)	21 (4.2)	18 (3.9)
M1 (%)	15 (1.1)	31 (9.0)	4 (0.8)	3 (0.6)
Treatment (%)				
RT	698 (53.2)	19 (5.5)	292 (58.1)	47 (10.2)
RT + C	585 (44.6)	121 (35.2)	201 (40.0)	154 (33.3)
Surgery + RT/C	10 (0.8)	138 (40.1)	0 (0.0)	229 (49.5)
Surgery	20 (1.5)	52 (15.1)	0 (0.0)	33 (7.1)
Unspecified curative treatment	0 (0.0)	14 (4.1)	10 (2.0)	0 (0.0)
UICC8 (%)				
I	587 (44.7)	58 (16.9)	97 (19.3)	66 (14.3)
II	260 (19.8)	63 (18.3)	222 (44.1)	224 (48.4)
III	206 (15.7)	50 (14.5)	105 (20.9)	87 (18.8)
IV	260 (19.8)	173 (50.3)	79 (15.7)	86 (18.6)

*RT* radiation therapy, *C* chemotherapy

### Statistics

Covariates available for adjustment are described in Table 1. Age was included as a continuous variable in the analyses, and the remaining variables were included as categorical variables. Overall survival (OS) was defined as the time from diagnosis of OPSCC to death from any cause. Progression was based on a biopsy or relevant imaging and progression-free survival (PFS) was defined as the time from diagnosis of OPSCC to time of progression at any site or death from any cause. Patients were censored at the last date of follow-up, or administratively censored 5 years after diagnosis. Kaplan–Meier curves were used to illustrate survival differences and significant differences were assessed with log-rank tests.

To evaluate which covariates influenced survival, we fitted multivariate Cox regression analyses with all factors in Table 1 except treatment included (full model), and ties were handled with the method suggested by Efron. Subsequently, we simplified the full models using a stepwise backward-elimination procedure with Akaike’s information criteria as stopping criteria (final model) using the R package rms and the function fastbw.^27^ All models are multivariable, i.e. factors are mutually adjusted, and thus the effect estimates cannot be interpreted marginally. In a subanalysis, we evaluated the effect of fitting a spline for age in the development model. This sub-analysis was for the nonlinear part of the spline considered as non-significant; e.g. OS (*p* = 0.92) and PFS (*p*  = 0.85).

To test whether the assumption of proportional hazards was violated, we tested for trends in the scaled Schoenfeld residuals of the final models.^28^ None of the final models violated the proportional hazards assumption. Based on the final models, nomograms were constructed to predict overall survival and PFS at 1, 3 and 5 years after diagnosis. We considered only complete cases (e.g. patients were excluded from the analysis in the case of missing information from one or more variables). *p* values less than 5% were considered significant and all analyses were performed in R version 3.0.3.^29^

### Validation and calibration of multivariate Cox regression models

We conducted external validation by applying our nomograms to the patient cohorts from Sweden, United Kingdom and Germany. We assessed nomogram model performance by examining overall accuracy (Brier score),^30^ calibration plots^31^ and discrimination (Harrell’s *C* index).^32^ In addition, we fitted a Weibull calibration model, as suggested by van Houwelingen and Putter, in which shifts in baseline cumulative hazard (obtained from the final Cox models), the effect of the prognostic index (the linear predictor in the Cox model) and the shape of the cumulative baseline hazard were tested.^33^ A smoothed version of the cumulative baseline hazard was used in the calibration model, where smoothing was done by linear interpolation.

## Results

### Population demographics

**Fig. 1:**
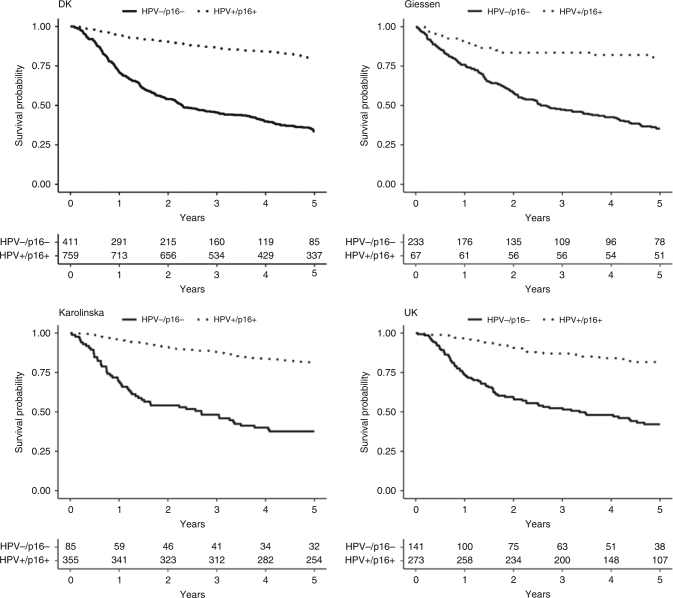
Kaplan–Meier curves depicting overall survival probability for HPV+/p16+ patients vs. HPV–/p16– patients

The development cohort consisted of 1313 patients with a total of 457 deaths (35%) during follow-up (Table 1). The majority of patients were males (72%), with a median age of 59.8 years at diagnosis, and had most frequently HPV+/p16+ tumours (58%). Patients typically presented with tumours in advanced nodal stage (78% with *N*+), with small primary tumours (68% T1 or T2), and in early UICC8 stage (65% UICC8 stage I or II). OS was for the HPV+/p16+ patients 95% (95% CI 93–96%) after 1 year, 86% (95% CI 84–89%) after 3 years and 80% (95% CI 77–83%) after 5 years of follow-up, and for the HPV–/p16– patients 71% (95% CI 65–74%) after 1 year, 46% (95% CI 41–51%) after 3 years and 34% (95% CI 29–39%) after 5 years (Fig. 1, Table 2). Demographic information and treatment modality for the validation cohorts are shown in Table 1.

**Table 2:** Overall survival estimates HPV+/p16+ and HPV–/p16– patients

	Eastern Denmark	Karolinska, Sweden	Giessen, Germany	The Predictr Consortium, UK
OS	HPV+/p16+	HPV+/p16+	HPV+/p16+	HPV+/p16+
1-year	95% (93–96%)	96% (94–98%)	91% (84–98%)	97% (95–99%)
3-year	86% (84–89%)	88% (85–91%)	84% (75–93%)	87% (83–91%)
5-year	80% (77–83%)	81% (77–85%)	81% (72–91%)	82% (77–87%)
OS	HPV–/p16–	HPV–/p16–	HPV–/p16–	HPV–/p16–
1-year	71% (67–75%)	69% (60–80%)	76% (71–82%)	74% (67–82%)
3-year	46% (41–51%)	48% (39–60%)	47% (41–54%)	52% (44–62%)
5-year	34% (29–39%)	38% (29–49%)	35% (30–42%)	42% (34–52%)
Censored cases	*n* = 888 (63.8%)	*n* = 389 (71.9%)	*n* = 164 (45.7%)	*n* = 420 (62.6%)

*OS* overall survival

### Overall survival

Five-year OS for the HPV+/p16+ patients in the Swedish cohort was 81% (95% CI 77–85%) and for the HPV–/p16– patients 40% (95% CI 31–52%); for the German cohort 81% (95% CI 72–91%) and 35% (95% CI 30–42%) and in the UK cohort it was 82% (95% CI 77–87%) and 42% (95% CI 34–52%) (Fig. 1, Table 2, Suppl. Table S1).

**Fig. 2:**
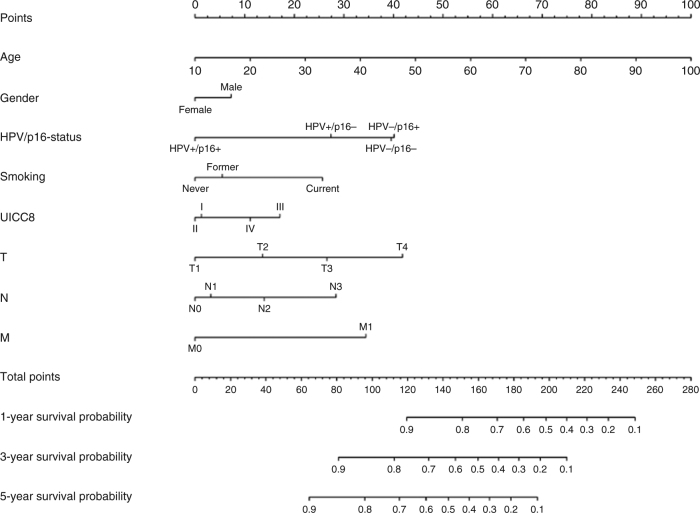
Predictive nomogram for overall survival. The nomogram is used by totalling the points identified on the top scale for each independent covariate. The total points scale is used to identify the probability of 1-, 3- and 5-year survival

The backwards elimination procedure left the model for overall survival unchanged, i.e. the model included age, gender, combined HPV and p16 status, smoking, T-, N-, and M-classification and UICC-8 staging (Table 3). The OS nomogram was used to predict the probability of death due to any cause at 1, 3 and 5 years after diagnosis (Fig. 2).

**Table 3:** Final model for overall survival in the development cohort (no covariates were removed from the full model)

	HR	2.5%	97.5%	*P*
Age	1.03	1.02	1.04	<0.01
Gender, Female (ref)				
Male	1.19	0.96	1.47	0.12
HPV–/p16– (ref)				
HPV–/p16+	1.01	0.63	1.64	0.96
HPV+/p16–	0.75	0.51	1.10	0.14
HPV+/p16+	0.39	0.26	0.58	<0.01
Smoking, Current (ref)				
Former	0.62	0.49	0.77	<0.01
Never	0.54	0.38	0.77	<0.01
UICC8 I (ref)				
UICC8 II	0.97	0.66	1.44	0.88
UICC8 III	1.46	0.92	2.31	0.11
UICC8 IV	1.27	0.63	2.56	0.51
T1 (ref)				
T2	1.39	1.02	1.87	0.03
T3	1.89	1.34	2.67	<0.01
T4	2.72	1.79	4.14	<0.01
N0 (ref)				
N1	1.08	0.81	1.43	0.59
N2	1.40	0.94	2.08	0.10
N3	1.98	1.29	3.04	<0.01
M0 (ref)				
M1	2.28	1.28	4.08	0.01

**Fig. 3:**
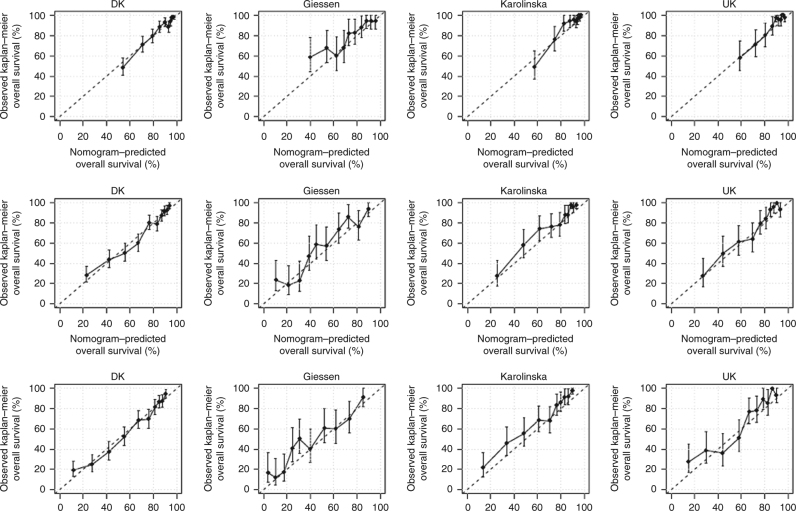
Calibration for 1-year (top row), 3-year (middle row) and 5-year overall survival. DK Denmark (development cohort)

Figure 3 shows the calibration plots for internal and external validation at 1, 3 and 5 years. The calibration model showed that the log cumulative baseline hazard was shifted by −0.51 (95% CI: −0.75 to –0.26) in the Swedish cohort, −0.35 (95% CI: −0.64 to −0.05) in the German cohort and −0.34 (95% CI: −0.62 to −0.07) in the UK cohort. The parameter regressing the log cumulative baseline hazard in the development cohort on the log cumulative baseline hazard in the German cohort was 0.83 (95% CI: 0.72–0.93) corresponding to a less-steep increase in the German cohort. For the Swedish and the UK cohort, the corresponding numbers were 0.91 (95% CI: 0.78–1.05) and 1.00 (95% CI: 0.85–1.15), respectively. Finally, the specification of the linear predictor for all three cohorts appeared correct with confidence intervals all including unity.

Harrell’s *C* index for the OS nomogram was 0.787 (95% CI 0.753–0.817), 0.772 (95% CI 0.747–0.817) and 0.766 (95% CI 0.746–0.788) for 1, 3 and 5 years, respectively. Similarly, external validation after 1, 3 and 5 years, gave *C*-indexes for the Swedish cohort of 0.836 (95% CI 0.775–0.881), 0.793 (95% CI 0.749–0.833) and 0.780 (95% CI 0.743–0.815); for the German cohort 0.712 (95% CI 0.655–0.764), 0.722 (95% CI 0.683–0.759) and 0.707 (95% CI 0.671–0.741) and for the UK cohort 0.815 (95% CI 0.775–0.864), 0.797 (95% CI 0.755–0.832) and 0.791 (95% CI 0.751–0.822). Brier plots for OS are presented in Suppl. Fig. S1 and histograms of the linear predictor plots are shown in Suppl. Fig. S2.

### Progression-free survival

**Fig. 4:**
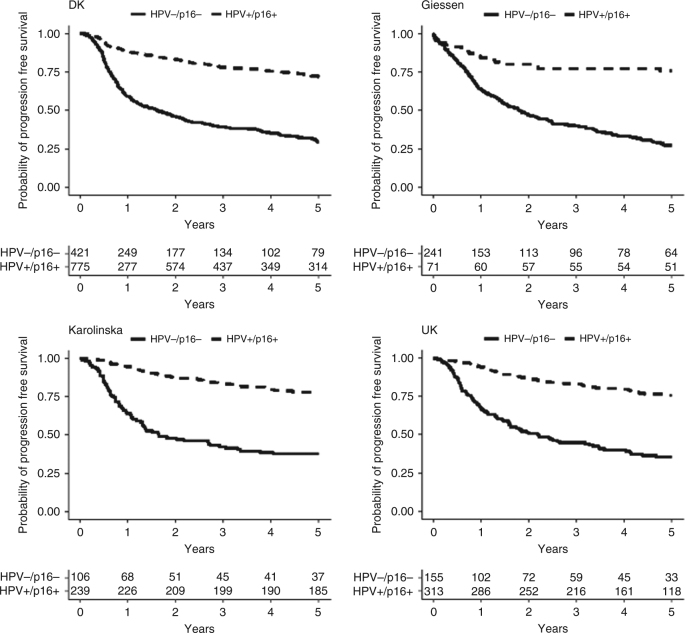
Kaplan–Meier curves depicting progression-free survival probability for HPV+/p16+ patients vs. HPV–/p16– patients

In total, 540 (41%) patients in the development cohort experienced disease progression or death, with 187 (24%) patients in the HPV+/p16+ subgroup vs. 274 (66%) patients in the HPV–/p16– subgroup (*p* < 0.001). Crude cumulative incidence of progression or death in the development cohort was at 5 years, 28% (95% CI 25–32%) for the HPV+/p16+ patient group and 71% (95% CI 66–75%) for the HPV–/p16– patient group (Fig. 4). In the validation cohorts, 208 (61%) of 344 patients in the German, 162 (32%) of 503 in the Swedish and 158 (34%) of 463 in the UK cohort developed disease progression or death. Follow-up times are given in Suppl. Table S1. In the multivariable model, the AIC backward- selection procedure led to exclusion of the variable UICC-8 staging and inclusion of the covariates age, gender, combined HPV and p16 status, smoking, T-, N- and M-classification (Table 4).

**Table 4:** Final model for progression-free survival in the development cohort (UICC-8 staging was removed from the full model)

	HR	2.5%	97.5%	*P*
Age	1.01	1.01	1.02	<0.01
Gender, Female (ref)				
Male	1.24	1.02	1.51	0.03
HPV–/p16– (ref)				
HPV–/p16+	0.91	0.65	1.26	0.57
HPV+/p16–	0.75	0.52	1.09	0.13
HPV+/p16+	0.41	0.33	0.52	<0.01
Smoking, Current (ref)				
Former	0.64	0.52	0.78	<0.01
Never	0.63	0.47	0.85	<0.01
T1 (ref)				
T2	1.43	1.09	1.87	0.01
T3	1.81	1.36	2.41	<0.01
T4	3.10	2.27	4.25	<0.01
N0 (ref)				
N1	1.17	0.92	1.50	0.20
N2	1.61	1.24	2.09	<0.01
N3	2.02	1.53	2.66	<0.01
M0 (ref)				
M1	1.79	1.02	3.13	0.04

**Fig. 5:**
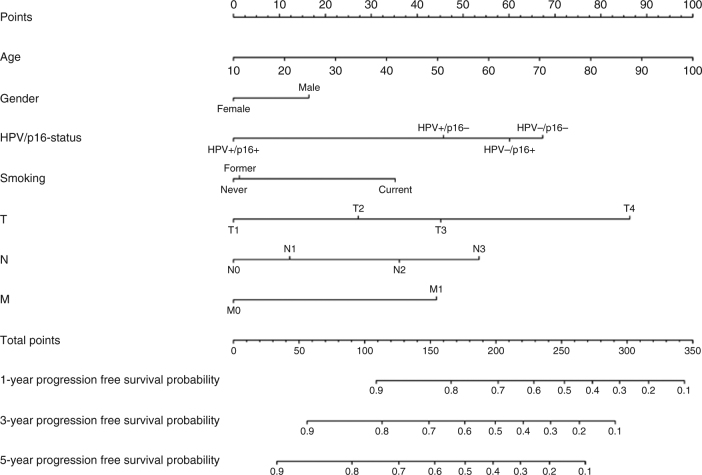
Predictive nomogram for progression-free survival. The nomogram is used by totalling the points identified on the top scale for each independent covariate. The total points scale is used to identify the probability of 1-, 3- and 5-year survival

**Fig. 6:**
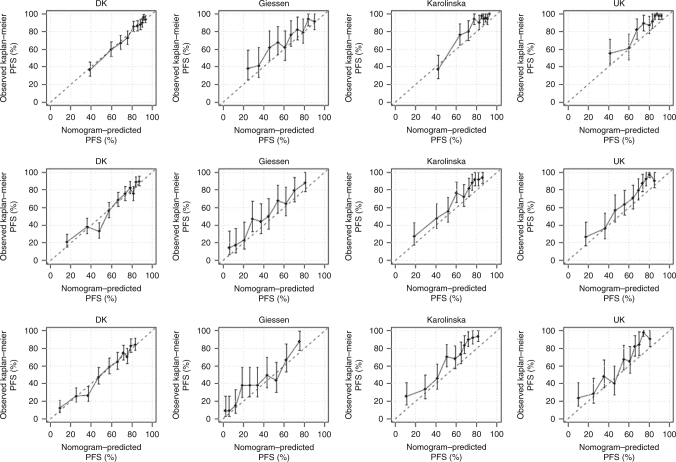
Calibration for 1-year (top row), 3-year (middle row) and 5-year progression-free survival. DK Denmark (development cohort) (PFS progression-free survival)

The nomogram for prediction of PFS at 1, 3 and 5 years is shown in Fig. 5. Harrell’s *C* index of the development cohort was 0.733 (95% CI 0.703–0.760), 0.728 (95% CI 0.704–0.750) and 0.725 (95% CI 0.703–0.747) at 1, 3 and 5 years. External validation of the nomogram for PFS after 1, 3 and 5 years, gave *C*-indexes of 0.805 (95% CI 0.745–0.852), 0.763 (95% CI 0.722–0.802) and 0.764 (95% CI 0.724–0.801), respectively, for the Swedish cohort; 0.714 (95% CI 0.663–0.761), 0.711 (95% CI 0.671–0.748) and 0.704 (95% CI 0.667–0.738), respectively, for the German cohort and 0.797 (95% CI 0.739–0.842), 0.778 (95% CI 0.735–0.812) and 0.771 (95% CI 0.731–0.805), respectively, for the UK cohort. The parameter regressing the log cumulative baseline hazard in the development cohort on the log cumulative baseline hazard in the German cohort was 0.88 (95% CI: 0.69–1.07), in the Swedish cohort 1.16 (95% CI: 0.97–1.36) and in the UK cohort 1.20 (95% CI: 0.99–1.40). The specification of the model for all cohorts appeared correct with confidence intervals all including unity. Calibration plots for internal and external validation of PFS are shown in Fig. 6, Brier plots in Suppl. Fig. S3 and histograms of the linear predictor plots in Suppl. Fig. S4.

## Discussion

This study presents multinational-validated nomograms for OS and PFS for patients with OPSCC. One of the main findings includes the identification of combined HPV-DNA and p16 status as an important and independent predictor for OS and PFS. The nomograms performed well in external validation across areas with high and low HPV prevalence. These models may facilitate discussions in clinical settings and aid in identifying lower-risk patients who could be candidates for de-escalation therapy, as well as higher-risk patients eligible for treatment-escalation trials. The online nomogram (www.orograms.org) can be used for more precise calculations than drawing lines on the nomogram.

The significance of the double biomarker can be exemplified in a typical patient case of a male, 60 years of age, nonsmoker, and classified as T2N2M0, UICC-8 stage II. If the tumour is HPV+/p16+, the 3- and 5-year OS estimates are 90% and 84%, respectively. However, if the tumour is HPV–/p16+, the 3- and 5-year OS estimates fall to 72% and 60%, respectively. Similar reductions are seen in PFS estimates when comparing HPV+/p16+ with HPV–/p16+ tumours. Although these numbers are estimates, they underline the importance of evaluating patient prognosis using the combined biomarker of HPV and p16.

Notably, HPV+/p16+ patients with T1–T2 and N1 tumours could be considered candidates for de-escalation therapy, as their survival is similar to the background population,^34^ and this might avoid some of the morbidity associated with therapy. Our models also encourage studies to better understand whether HPV+/p16+ patients with N2 and N3 tumours are eligible for de-escalation as well. Notably, at least nine de-escalation treatment trials are ongoing or finishing.^35^ Our nomograms are likely to be applicable to these and future trials, as we report similar 5-year survival or progression rates as in North America,^36–38^ Western,^39–41^ Southern,^42^ and Northern Europe,^2,43^ Australia^44^ and China.^45^

One of the strengths in this study is the joint use of HPV and p16 for scoring tumours. Other advantages are the large sizes of the development and validation cohorts, all from areas with universal, tax-financed health-care systems diminishing selection bias. In a previous smaller study in a region with very low HPV prevalence (<20%), the development cohort was not from a population-based, non-selected setting when constructing nomograms for OS and PFS in OPSCC patients.^17^ A recently published nomogram from the United States, also with smaller cohorts, mainly included patients from private hospitals, and did not include the important double biomarker of HPV and p16.^18^ This study also had difficulties in showing the significance of p16 alone for, e.g. PFS.

In this study, we chose overall survival instead of disease-specific survival as a primary endpoint because it represents the cumulative effect of competing diseases, treatment morbidity and age on patient survival. As disease progression is associated with significantly poorer outcome and consequently a decrease in quality of life, we developed a nomogram with PFS as the endpoint. The PFS nomogram therefore complements the overall survival nomogram well.

Although our training cohort is population based and selection bias is minimised, the nomograms have limitations. With respect to accuracy, the CIs at the various predicted probabilities of recurrence should be considered if using these nomograms in clinical settings. The final models performed well in calibration and discrimination, but the level (risk of outcomes) is––as expected––not identical across cohorts. This is most evident in the German cohort, and this risk should be taken into account when using the models. The German cohort might perform worse due to several factors; partly a significantly lower HPV prevalence, higher smoking, higher share of patients who experience progression and a greater share of patients in advanced stage (e.g. stage 3 and 4). Although this is adjusted for, it should be considered whether these nomograms are best suitable in HPV-high-risk areas. A possible other bias in this model is the number of censored patients, as observed in the crude-survival analysis. The Danish, UK and Swedish centres have ~60–70% censorship opposed to the German with merely 45% censored patients.

These nomograms are only applicable for patients who underwent evaluation at multidisciplinary head and neck cancer centres, as performance of the nomograms is likely to be worse for patients who do not attend multidisciplinary evaluation.

p16 overexpression is a standard surrogate biomarker for high-risk HPV-associated OPSCC.^23,46,47^ But notably, p16 overexpression is also present in a number of non-HPV-driven tumours probably related to RAS and BRAF mutations^48^ although not in KRAS.^49^ Other head and neck carcinomas have proven to be HPV–/p16+ likely related to misconfigurations in the p16–Rb–cyclin-D1 pathway inducing cell cycle activation in HPV-negative carcinomas.^50^

The prevalence of HPV-associated OPSCC ultimately depends on the sensitivity and specificity detection method employed, and using different methods between cohorts as in this study, might lead to discordant results in HPV/p16 testing. All four centres employed different p16 and HPV-testing tools, which is a potential shortcoming. All methods cover the most relevant HR-HPV types (HPV 16, 18, 31, 33, 35, 39, 45, 51, 52, 56, 58, 59 and 66). However, the Ventana in situ method (UK cohort) is highly sensitive but less specific opposed to amplification of HPV DNA by general primers (GP5+/6+) with presumed high sensitivity and specificity (Danish, Swedish and German cohort), and the subsequent detection of the PCR products with type-specific probes, e.g. bead-based multiplex, might differ. A limitation of this study is also the use of different p16 antibodies across centres, potentially leading to a discrepancy in p16 positivity. Notably, more than 90% of all tumours in this study are examined with the use of the same antibody (clone E6H4). Preferably, a subset of tumours should be tested with all methods to uncover potential shortcomings.

In conclusion, we developed and validated nomograms for OPSCC patients and known HPV-DNA and p16 status. The nomograms are applicable for both high and low HPV areas. Combining HPV-DNA and p16 status is essential for accurate prognostication. Future work might focus on validating our results and incorporating additional prognostic factors, including nomograms specific for salvage treatment for relapsed disease, as well as including outcome measures which have shown to influence outcome (i.e. weight loss, education and anaemia) and outcomes such as histopathological evaluations.

## Electronic supplementary material

Supplementary

## Availability of data and materials

Information on data supporting the results is available from the corresponding author by special request.
