# Supplementary material for: Development and external validation of nomograms in oropharyngeal cancer patients with known HPV-DNA status: a European Multicentre Study (OroGrams)
Source: Br J Cancer. 2018 May 24;118(12):1672–81. doi: 10.1038/s41416-018-0107-9 (PMC6008433; doi:10.1038/s41416-018-0107-9)

**Supplementary Tables**

Suppl. Table S1 Number of patients, events, and median survival for overall- and progression-free survival and corresponding confidence interval. Blank boxes mean that the probability of survival is greater than 50% after 5 years, which implies that the median is not defined. Numbers are with censoring after 5 years.

|  |  | **Overall survival** | | | | |
| --- | --- | --- | --- | --- | --- | --- |
|  |  | Records | events | median | 0.95 LCL | 0.95 UCL |
| Eastern Denmark | HPV-/p16- | 411 | 257 | 2.29 | 1.95 | 3.05 |
| Giessen, Germany | HPV-/p16- | 233 | 149 | 2.56 | 2.09 | 3.86 |
| Karolinska, Sweden | HPV-/p16- | 85 | 53 | 2.68 | 1.39 | 4.08 |
| UK | HPV-/p16- | 141 | 75 | 3.35 | 2.18 |  |
| Eastern Denmark | HPV+/p16+ | 759 | 131 |  |  |  |
| Giessen, Germany | HPV+/p16+ | 67 | 13 |  |  |  |
| Karolinska, Sweden | HPV+/p16+ | 355 | 67 |  |  |  |
| UK | HPV+/p16+ | 273 | 44 |  |  |  |
|  |  | **Progression-free survival** | | | | |
|  |  | Records | events | median | 0.95 LCL | 0.95 UCL |
| Eastern Denmark | HPV-/p16- | 411 | 274 | 1.57 | 1.19 | 2.08 |
| Giessen, Germany | HPV-/p16- | 233 | 167 | 1.86 | 1.48 | 2.48 |
| Karolinska, Sweden | HPV-/p16- | 85 | 54 | 1.51 | 1.07 | 3.77 |
| UK | HPV-/p16- | 141 | 85 | 2.21 | 1.59 | 3.59 |
| Eastern Denmark | HPV+/p16+ | 759 | 187 |  |  |  |
| Giessen, Germany | HPV+/p16+ | 67 | 15 |  |  |  |
| Karolinska, Sweden | HPV+/p16+ | 355 | 80 |  |  |  |
| UK | HPV+/p16+ | 273 | 53 |  |  |  |

**Supplementary figures**

Suppl. Figure S1 Brier score (plots) for overall survival.


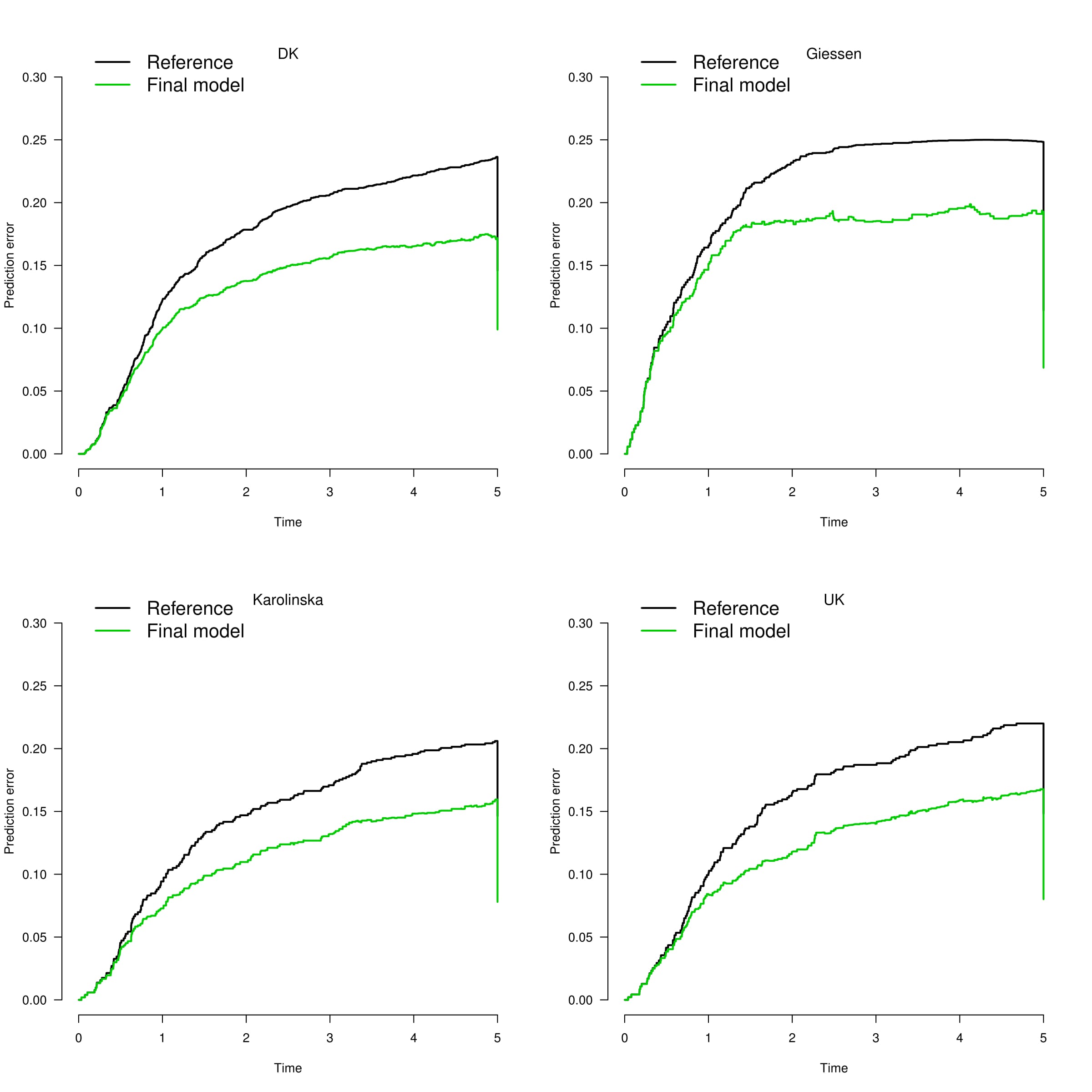


Suppl. Figure S2 Linear predictor for overall survival


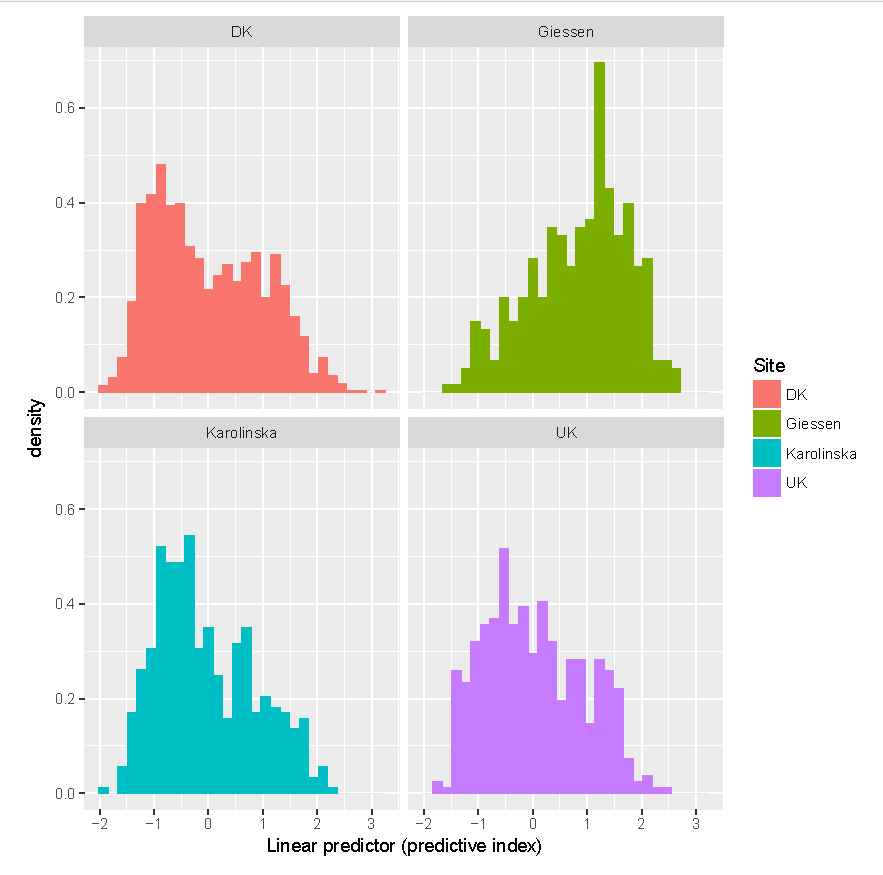


Suppl. Figure S3 Brier score (plots) for progression-free survival


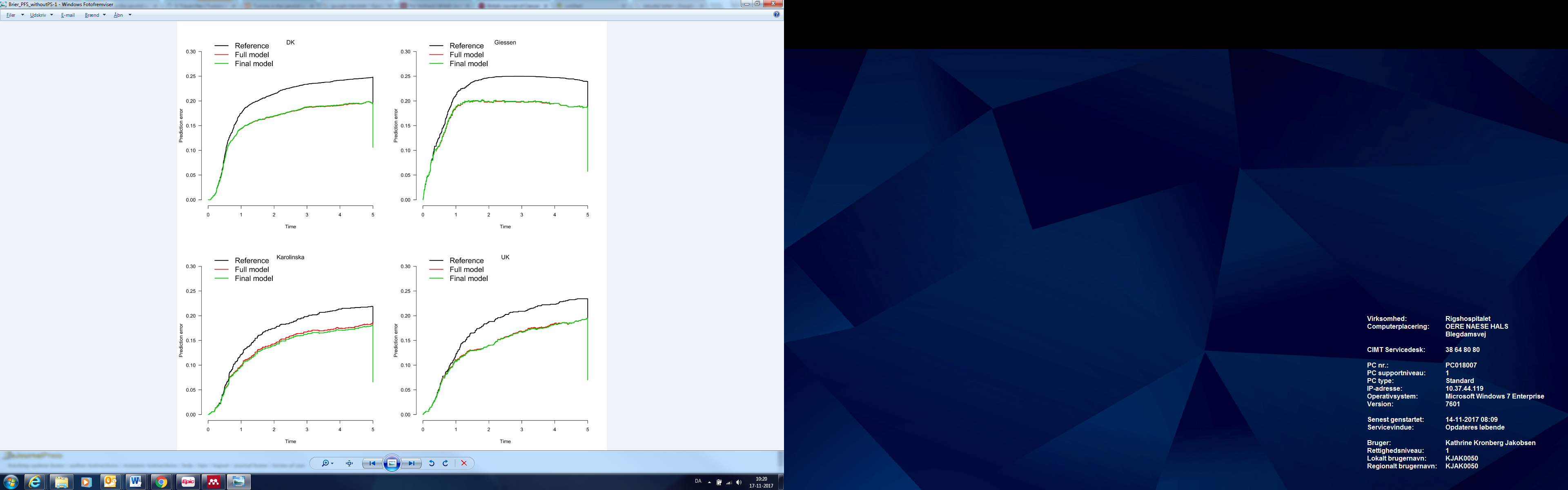


Suppl. Figure S4 Linear predictor for progression-free survival


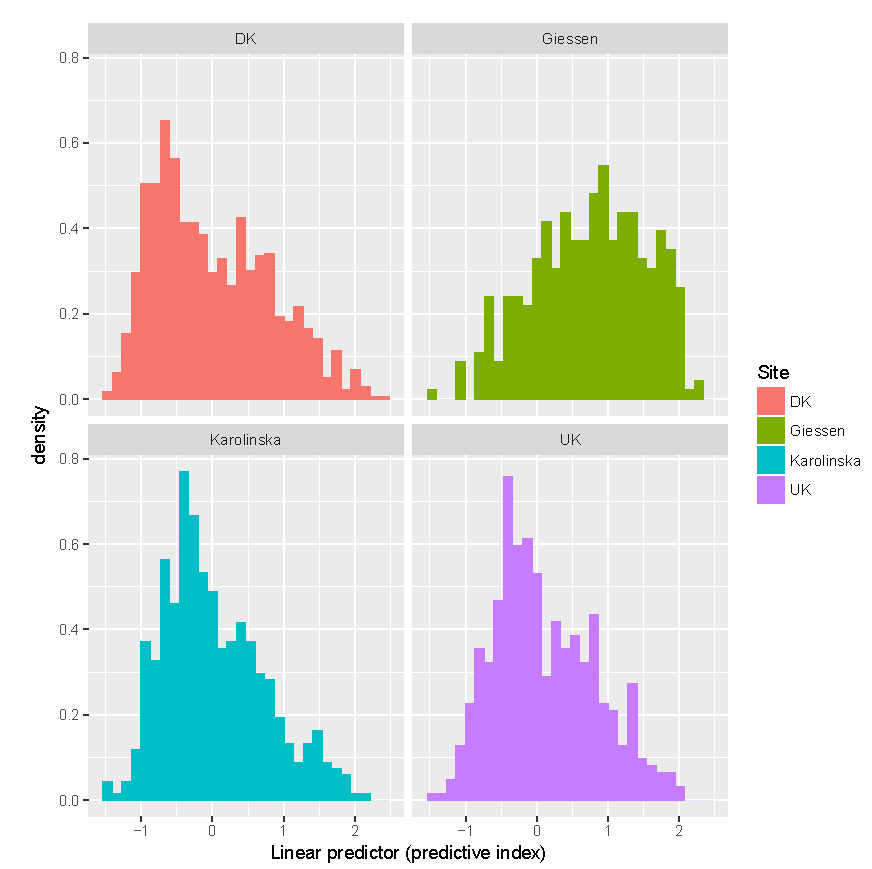

Supplement: Supplementary file 1 — Supplementary [file 41416_2018_107_MOESM1_ESM.docx]
